# Diagnosis and prognosis of myocardial infarction on a plasmonic chip

**DOI:** 10.1038/s41467-020-15487-3

**Published:** 2020-04-03

**Authors:** Wei Xu, Lin Wang, Ru Zhang, Xuming Sun, Lin Huang, Haiyang Su, Xunbin Wei, Chia-Chun Chen, Jiatao Lou, Hongjie Dai, Kun Qian

**Affiliations:** 1 School of Biomedical Engineering and Med-X Research Institute, Shanghai Jiao Tong University, Shanghai, 200030 China; 2 State Key Laboratory for Oncogenes and Related Genes, Division of Cardiology, Renji Hospital, School of Medicine, Shanghai Jiao Tong University, 160 Pujian Road, Shanghai, 200127 China; 3 Department of Laboratory Medicine, Shanghai Chest Hospital, Shanghai Jiao Tong University, Shanghai, 200030 China; 4 Department of Chemistry, National Taiwan Normal University, Taipei, 11677 Taiwan; 5 Department of Chemistry, Stanford University, Stanford, CA 94305 USA

**Keywords:** Biomarkers, Myocardial infarction, Surface chemistry, Biomedical engineering

## Abstract

Cardiovascular diseases lead to 31.5% of deaths globally, and particularly myocardial infarction (MI) results in 7.4 million deaths per year. Diagnosis of MI and monitoring for prognostic use are critical for clinical management and biomedical research, which require advanced tools with accuracy and speed. Herein, we developed a plasmonic gold nano-island (pGold) chip assay for diagnosis and monitoring of MI. On-chip microarray analysis of serum biomarkers (e.g., cardiac troponin I) afforded up to 130-fold enhancement of near-infrared fluorescence for ultra-sensitive and quantitative detection within controlled periods, using 10 μL of serum only. The pGold chip assay achieved MI diagnostic sensitivity of 100% and specificity of 95.54%, superior to the standard chemiluminescence immunoassay in cardiovascular clinics. Further, we monitored biomarker concentrations regarding percutaneous coronary intervention for prognostic purpose. Our work demonstrated a designed approach using plasmonic materials for enhanced diagnosis and monitoring for prognostic use towards point-of-care testing.

## Abstract (web summary)

The gold standard for diagnosis and monitoring of myocardial infarction is a chemiluminescence assay based on the detection of cardian troponin I (cTnI). Here, the authors develop a plasmonic gold nano-island chip assay for ultrasensitive detection of cTnI in as little as 10 μL of serum.

## Introduction

While cardiovascular diseases (CVDs) threaten human health with the lifetime risk exceeding 30%, myocardial infarction (MI) is the leading cause of death for CVDs nowadays^1,2^. Notably, the influence of MI is becoming more severe in recent decades with an annual growth rate of over 3.6%^3,4^, due to changes in human behavior and lifestyle, particularly in developing countries^5,6^. Considering the substantial progress in the treatment of MI, current management of MI is focused on diagnostic and prognostic approaches, which can reduce the medical costs and raise the survival ratio as well as life quality of patients^7,8^. Therefore, an advanced analytical tool would be of key significance toward efficient management of MI.

Blood test serves as the major clinical method for monitoring of diseases including but not limited to MI^9,10^. Blood tests are convenient, noninvasive, and accurate, superior to the conventional methods such as the electrocardiogram (ECG) and physical examination (PE)^11,12^. Nevertheless, the effectiveness of blood tests in clinics relies on designed materials and device, considering the following aspects: (I) high sensitivity and specificity for precision diagnostics; (II) low sample volume for point-of-care testing (POCT); (III) disease-specific performance with tailored analytical features (e.g., speed and selection of biomarkers); and (IV) multi-functions in clinical application, such as monitoring for prognostic use^13,14^. To date, the present blood tests are still far from ideal for monitoring of MI and call for new platforms addressing the above aspects.

Plasmonic materials are usually noble metals and afford unique optical properties for diverse biomedical use^15–17^. Particularly, near-infrared fluorescence enhanced (NIR-FE, the wavelength of 650–1700 nm) detection promises the state-of-the-art in vivo imaging and in vitro diagnosis, through a series of pre-selected materials interfaces^18–20^. For example, given characteristic gold nano-islands and immobilized immunoassays on the surface, plasmonic gold nano-island (pGold) chips afford NIR-FE detection of target molecules by high-performance microarrays^21^. NIR-FE detection has engaged in diagnostics including cancer^22^, diabetes^23,24^, hypertensive heart disease^25^, and infectious diseases etc^26^. To date, the application of NIR-FE in MI has not been established and a further obstacle is monitoring for prognostic use in clinics, both of which should be tackled due to the urgent needs for better MI management and multi-use in clinical laboratory and POCT.

Herein, a plasmonic gold nano-island chip based assay was developed for the diagnosis and monitoring of MI. On-chip microarray analysis of serum biomarkers (e.g., cardiac troponin I) afforded up to 130-fold enhancement of near-infrared fluorescence for ultrasensitive and quantitative detection within controlled periods (down to 30 min), using 10 μL of serum only. We achieved MI diagnostic sensitivity of 100% and specificity of 95.54% with area under the curve (AUC) of 0.976 (95% confidence interval (CI): 0.950–1.000, *p* = 2.85 × 10^−11^), superior to the standard chemiluminescence immunoassay in cardiovascular clinics. We performed post-operative monitoring of percutaneous coronary intervention (PCI, the universal treatment procedure for MI). Our work demonstrated a designed approach using plasmonic materials for enhanced diagnosis and monitoring for prognostic use toward point-of-care testing.

## Results

### Construction and characterization of pGold chip assay

**Fig. 1:**
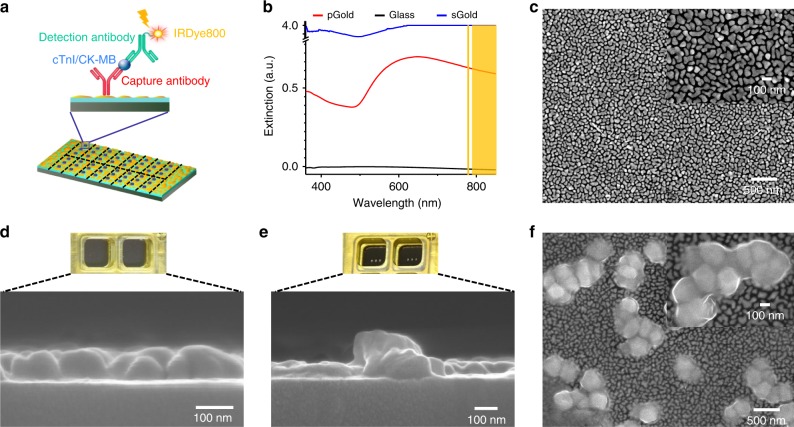
Construction and characterization of antibody-printed pGold chips. **a** Schematic illustration of antigen (cTnI/CK-MB) detection on pGold chip using a sandwich assay. **b** Extinction spectra of pGold chip (red line), glass (black line), and sGold chip (blue line) overlaid with the excitation and emission (yellow shaded area) regions of IR800 dye. **c** Top-view SEM of pGold chip (*n* ≥ 3 randomly selected). **d** Digital image of two bulk reaction wells and side-view SEM of gold islands on pGold chip. **e** Digital image of two antibody-printed reaction wells and side-view SEM of capture antibodies on pGold chip. **f** Top-view SEM of capture antibodies on pGold chip (*n* ≥ 3 randomly selected). Insets of (**c**) and (**f**) were the zoomed SEM images. Source data are provided as a Source Data file.

The sandwich immunoassay was constructed toward selected biomarkers on pGold chips (Fig. 1a). pGold chips (obtained from Nirmidas Biotech Inc.) afforded unique extinction spectra (Fig. 1b) and discontinuous gold nano-islands on the surface for NIR-FE detection (Fig. 1c). As shown in the high-resolution top-view SEM image inset of Fig. 1c, there are gold nano-islands with size of 80–200 nm and gaps of 10–30 nm. The wavelength of the plasmon band was ~610 nm for plasmonic gold nano-island chip and ~570 nm for sputter gold (sGold) chip^21,27^. We printed the triplicate microarrays of antibodies on the pGold chip (digital images in Fig. 1d, e) and assembled the device capable of 16 samples detection in parallel (Supplementary Fig. 1) for high throughput application.

The pGold chips had a surface layer of gold with a thickness of ~80 nm and microarray printing introduced the additional layer of antibodies, according to the cross-section SEM in Fig. 1d, e. The distribution of antibodies aggregates on the surface was revealed by the top-view SEM in Fig. 1f, consistent with the cross-section SEM. Therefore, using the as-printed pGold chips to quantitate serum biomarkers, immunoassays can be conducted within the optimized proximity to plasmonic surface (~hundreds of nanometers)^21,22^ for NIR-FE detection.

### NIR-FE detection of serum biomarkers on-chip

**Fig. 2:**
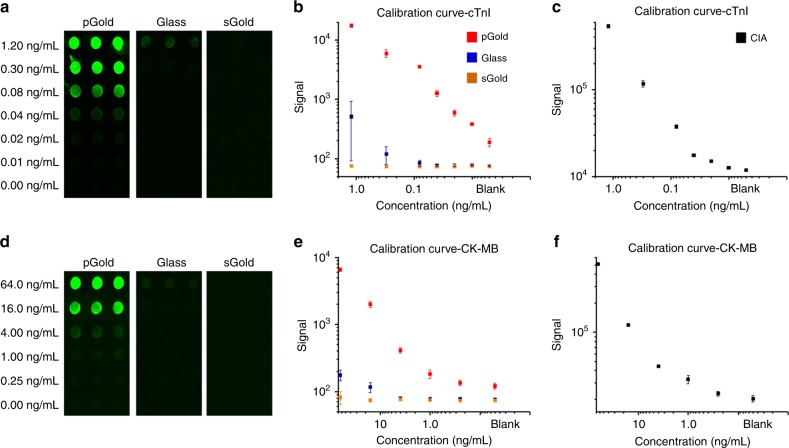
Near-infrared fluorescence enhanced microarray detection of biomarkers. **a** Fluorescence mapping results by IRDye800 labeled detection of cTnI (0–1.2 ng mL^−1^) on pGold (left), glass (middle), and sGold (right) chips. **b** Calibration curves comparing detection limits and dynamic ranges for cTnI on pGold (red), glass (blue), and sGold (yellow) chips. We performed three independent experiments on pGold, glass, and sGold chips to calculate the standard deviation (s.d.) as error bars and data are shown as the mean ± s.d. (*n* = 3). **c** Calibration curve of cTnI quantification by CIA. We performed three independent CIA experiments and data are shown as the mean ± s.d. (*n* = 3). Similarly, **d** fluorescence mapping results, **e** calibration curves comparing detection limits and dynamic ranges by different chips, and **f** calibration curve by CIA were obtained for CK-MB. We performed three independent experiments and data are shown as the mean ± s.d. (*n* = 3). All samples used for the calibration curves were standards containing known concentrations of biomarkers provided by the vendor (Tellgen). Source data are provided as a Source Data file.

The pGold chips enhanced NIR fluorescence up to 130-fold in the detection of cardiac troponin I (cTnI), compared with the glass chips according to fluorescence mapping results (Fig. 2a, Supplementary Tables 1, 2). The sGold chips with continuous gold film afforded reduced fluorescence due to the surface quenching effect^28^. For glass chips, the silica glass afforded no fluorescence enhancement due to non-plasmonic resonances of bulk silicate^29,30^ and only weak signals of fluorophore IRDye800 with a quantum yield of 0.05–0.1%^21,31^ can be observed, for 0.3 ng mL^−1^ cTnI with average fluorescence intensity of 43.84 after background noise subtraction. For sGold chips, the continuous gold film resulted in a decrease of quantum yield and quenching of fluorescence^32,33^, for 0.3 ng mL^−1^ cTnI with average fluorescence intensity of ~0 after background noise subtraction. For pGold chips, the discontinuous gold nano-islands produced surface plasmon resonance under laser^22,25^ and the enhanced near-infrared fluorescence can be obtained, for 0.3 ng mL^−1^ cTnI with average fluorescence intensity of 5727.22 after background noise subtraction. Consequently, near-infrared fluorescence enhanced detection can be obtained on pGold chips, compared with the glass chips (*p* < 0.0001) and sGold chips (*p* < 0.0001), respectively. Therefore, only the gold nano-island platform (pGold chips) yielded the expected results.

Calibration curves were recorded for cTnI in a dynamic range of 0.01–1.20 ng mL^−1^ on the pGold chips (Fig. 2b, superior to glass and sGold chips), with limit-of-detection (LOD) and limit-of-quantification (LOQ) of 0.0100 and 0.0157 ng mL^−1^, respectively (Table 1). The results demonstrated the improved LOD and LOQ of pGold chip in detecting cTnI (*p* < 0.05), compared with the chemiluminescence immunoassay (CIA, the current gold standard in serum biomarkers based MI diagnosis) in Fig. 2c, Table 1, and Supplementary Table 1. Specifically, we calculated the LOD of pGold chip assay using the following Eq. (1):

1 $$\left[ {{\mathrm{LOD}}} \right] =	 \, \left[ {{\mathrm{mean}}\;{\mathrm{blank}}\;{\mathrm{value}}} \right] + 3 \times \left[ {\mathrm{standard}}\;{\mathrm{deviation}}\;\left( {{\mathrm{SD}}} \right)\right.\\ 	\,{\mathrm{of}}\; {\mathrm{the}}\; \left.{\mathrm{blank}}\;{\mathrm{value}} \right]$$

which had been reported in previous literatures^22,23,26^.

**Table 1:** LODs, LOQs, and cutoffs using pGold chip and CIA.

	cTnI (ng mL^−1^)	CK-Mb (ng mL^−1^)
CIA-LOD^a^	0.0152	1.3245
CIA-LOQ^b^	0.0208	2.8393
CIA-cutoffs	0.04	4.00
pGold-LOD^a^	0.0100	0.2715
pGold-LOQ^b^	0.0157	1.1348
pGold-cutoffs	0.03	3.13

^a^LODs were calculated by applying the mean blank value plus 3 times of s.d. to the fitting line of calibration curves.

^b^LOQs were calculated by applying the mean blank value plus 10 times of s.d. to the fitting line of calibration curves.

For comparison, the LOD in high-sensitivity cTnI assays was calculated using the following Eqs. (2) and (3):

2 $$\left[ {{\mathrm{LOD}}} \right] =	 \, \left[ {{\mathrm{limit{\hbox {-}}of{\hbox {-}}blank}}\;\left( {{\mathrm{LOB}}} \right)} \right] + 1.645 \times \left[{\mathrm{SD}}\;{\mathrm{of}}\;{\mathrm{the}}\, {\mathrm{lowest}}\right.\\ 	\left.\,{\mathrm{measurable}}\;{\mathrm{cTnI}}\;{\mathrm{value}}\;{\mathrm{(0}}{\mathrm{.01}}\,{\mathrm{ng}}\,{\mathrm{mL}}^{{\mathrm{ - 1}}}\; {\mathrm{in}}\;{\mathrm{our}}\;{\mathrm{work)}}\right]$$

3 $$\left[ {{\mathrm{LOB}}} \right] = \left[ {{\mathrm{mean}}\;{\mathrm{blank}}\;{\mathrm{value}}} \right] + 1{\mathrm{.645 \times }}\left[ {{\mathrm{SD}}\;{\mathrm{of}}\;{\mathrm{the}}\;{\mathrm{blank}}\;{\mathrm{value}}} \right]$$

which was also reported in previous literatures^34–36^.

Notably, the LOD of pGold chip assay was calculated to be 0.0030 ng mL^−1^ (3 ng L^−1^) based on the same equation as high-sensitivity cTnI assays (Eq. (2)), comparable to the LOD of current high-sensitivity cTnI assays (1–5 ng L^−1^). Moreover, the cutoffs of current high-sensitivity cTnI assays are set to be 14–52 ng L^−1^ (refs. ^37–39^), even for 0/1h-algorithm or 0/2h-algorithm, according to the guidelines of commercial diagnostic kits and literature reports, to avoid false positives and achieve high diagnostic specificity (>95%). Therefore, the LOD of pGold chip assay (3 ng L^−1^) is comparable to the LOD of current high-sensitivity cTnI assays (1–5 ng L^−1^) and sufficient for clinical use considering the recommended cutoffs (14–52 ng L^−1^).

Sensitivity is fundamentally important for the detection of serum biomarkers for efficient diagnosis of MI, due to the low concentration of biomarkers in the early stages of the disease. Similar results were obtained through NIR-FE detection of creatine kinase isoenzyme MB (CK-MB, Fig. 2d) on the pGold chips, showing advantages of pGold as a platform technology (Fig. 2e, f, Table 1, Supplementary Table 3). Notably, NIR-FE of cTnI and CK-MB on pGold chips yielded coefficient variation < 15% (Supplementary Tables 4, 5) in the calibration curve for low concentrations (0.01–1.20 ng mL^−1^ for cTnI and 0.25–64.00 ng mL^−1^ for CK-MB, Supplementary Tables 1, 3), validating the reproducibility and accuracy of our assay for clinical use.

### Diagnosis of MI patients by serum biomarkers

We detected 10 μL of serum samples from 112 MI patients (75 males and 37 females, with median age of 64.39, who were diagnosed with MI and without other major diseases on the basis of symptoms of myocardial ischemia, elevated cardiac biomarker, the electrocardiogram (ECG), and angiography) and 112 healthy controls (81 males and 31 females with median age of 61.66, who were diagnosed without MI and other major diseases) by the pGold chip assay for diagnosis of MI (Supplementary Table 6). Patients/controls known to have other medical conditions (such as active bleeding) were excluded. Both age and gender were matched with *p* > 0.05 (Supplementary Table 6, according to Student’s *t*-test and Fisher’s exact test), between the MI patients and healthy controls.

**Fig. 3:**
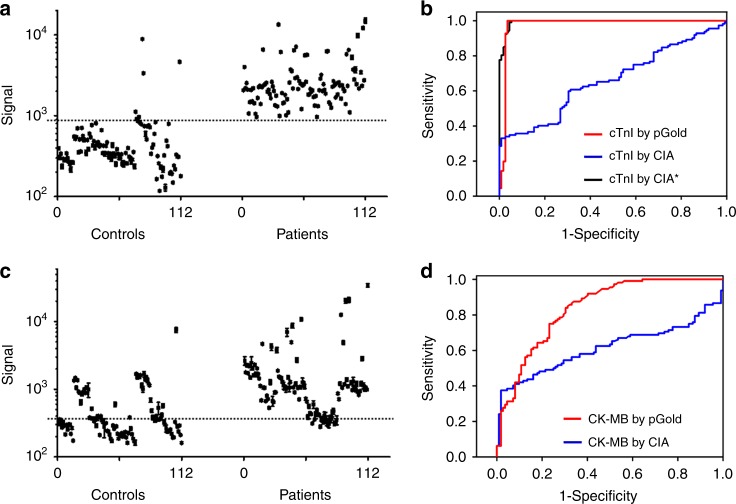
Serum tests for diagnosis of MI using cTnI and CK-MB. **a** Signal quantification on pGold chips for the detection of 112 MI patients and 112 controls using cTnI. All experiments were conducted with *n* = 3; mean ± s.d. **b** ROC curves for diagnosis by cTnI. The red and blue lines represented pGold chips and CIA, respectively. The black line (CIA*) represented the CIA based on the Abbott Architect assay. **c** Signal quantification on pGold chips for the detection of MI patients and controls using CK-MB. All experiments were conducted with *n* = 3; mean ± s.d. **d** ROC curves for diagnosis by CK-MB. The red and blue lines represented pGold chips and CIA, respectively. The dashed lines were the intensity cutoffs corresponding to serum concentrations of 0.03 ng mL^−1^ for cTnI and 3.13 ng mL^−1^ for CK-MB. Source data are provided as a Source Data file.

According to the fluorescence intensity from patients and controls (NIR-FE detection in Fig. 3a, CIA results in Supplementary Fig. 2a), the corresponding receiver operating characteristic (ROC) curves of cTnI were plotted for MI diagnosis (Fig. 3b). The pGold chip assay and CIA afforded AUC of 0.976 (95% CI: 0.950–1.000, *p* = 2.85 × 10^−11^) and 0.662 (95% CI: 0.590–0.734, *p* = 0.000023), respectively, in Table 2. The serum samples were diluted to the same volume (90 μL) by fetal bovine serum (FBS) for both plasmonic sensing platform and conventional approach (CIA), to test these two different methods under the same sample conditions. We used the CIA on the UniCel DxI 800 platform (Beckman Coulter), which is a reported high-sensitivity cTnI assay in clinic use according to current guidelines^40^ and requires 200 μL of serum for the optimal standard of care use. The decrease of sample volume (10 μL of serum in this work) resulted in the poor performance of the test. We used the same sample volume (10 μL of serum) for both CIA and pGold chip assays, to make a valid comparison.

**Table 2:** Sensitivity and specificity with AUCs and CI using pGold chip and CIA.

	CIA^a^	pGold^b^
Sensitivity-cTnI	59.82%	100%
Specificity-cTnI	70.54%	95.54%
AUC-cTnI^c^	0.662	0.976
95% CI^d^	0.590–0.734	0.950–1.000
*p* value-cTnI^e^	0.000023	2.85 × E^−11^
Sensitivity-CK-MB	24.11%	86.61%
Specificity-CK-MB	98.21%	66.07%
AUC-CK-MB^c^	0.614	0.821
95% CI^d^	0.537–0.692	0.786–0.886
*p* value-CK-MB^e^	0.000493	0.000338

^a^Sensitivity and specificity by CIA were obtained at the cutoffs of 0.04 ng mL^−1^ for cTnI and 4 ng mL^−1^ for CK-MB.

^b^Sensitivity and specificity by pGold chip were obtained at the lowered cutoffs of 0.03 ng mL^−1^ for cTnI and 3.13 ng mL^−1^ for CK-MB.

^c^AUCs were obtained using the SPSS software (version 19.0, SPSS Inc., Chicago).

^d^CI was calculated based on the Clopper–Pearson method.

^e^*p*-Value was calculated based on the two-sided Student’s *t*-test.

Superior to CIA with diagnostic sensitivity of 59.82% and specificity of 70.54%, the pGold chip assay afforded markedly improved diagnostic sensitivity of 92.86% and specificity of 97.32%, based on the recommended cutoff concentration by CIA (0.04 ng mL^−1^ for cTnI, Tables 1, 2)^41–43^, due to the enhanced LOD and LOQ. Notably, we achieved further enhanced diagnostic sensitivity of 100% and specificity of 95.54%, based on the lowered cutoff concentration by pGold (0.03 ng mL^−1^ for cTnI, Fig. 3, Tables 1, 2), which is sufficient to be of clinical use and comparable to Food and Drug Administration (FDA) approved assays for MI (with sensitivity > 95% and AUC > 0.96)^44,45^. We have also added the results using 200 μL of serum for cTnI detection by CIA (Supplementary Fig. 3, with diagnostic sensitivity of 100% and specificity of 96.43% based on the lowered cutoff concentration of 0.03 ng mL^−1^ for cTnI), as the supporting data for the direct comparison. Also, we used Abbott Architect kit and i2000SR platform (consuming 200 μL of serum), which is a reported high-sensitivity cardiac troponin I (hs-cTnI) assay according to the current guidelines^37,40,46^. The hs-cTnI assay (Architect) afforded the diagnostic sensitivity of 100%, specificity of 94.64%, and AUC of 0.994 (95% CI: 0.987–1.000) based on the cutoff concentration of 0.03 ng mL^−1^ (CIA* in Fig. 3b, Supplementary Fig. 4). Therefore, the pGold chip platform achieved high-sensitivity cTnI detection using 10 μL of serum only, compared with CIA using 200 μL of serum. And the diagnostic performances of two assays were comparable. The success can be due to the highly sensitive troponin testing based on the plasmonic platform with NIR-FE detection, which is highly sensitive and accurate even at very low concentrations.

In parallel, the diagnostic performance of CK-MB (Fig. 3c, Supplementary Fig. 2b) was studied and the corresponding ROC curves were recorded (Fig. 3d). Limited AUC of 0.821 (95% CI: 0.786–0.886, *p* = 0.000338, sensitivity of 86.61% and specificity of 66.07%) was obtained for CK-MB using the pGold chip assay superior to CIA with AUC of 0.614 (95% CI: 0.537–0.692, *p* = 0.000493, sensitivity of 24.11% and specificity of 98.21%), based on the recommended cutoff concentration by CIA (4 ng mL^−1^ for CK-MB, Tables 1, 2). Still unsatisfactory diagnostic sensitivity of 86.61% and specificity of 66.07% were obtained using the pGold chip assay, based on the lowered cutoff concentration by pGold (3.13 ng mL^−1^ for CK-MB, Fig. 3, Tables 1, 2), which was consistent with previous literatures^47,48^ and confirmed the key role of cTnI over CK-MB in the diagnosis of MI. Therefore, it was demonstrated that NIR-FE detection of selected biomarkers (cTnI) by the pGold chip assay achieved high diagnostic capability (AUC of 0.976) with a low sample volume of 10 μL of serum only, in screening of MI toward potential POCT, which is consistent with previous literature and confirms the key role of cTnI in the diagnosis of MI.

The high specificity of >95% referred to the diagnostic specificity, which can be attributed to both the rational selection of biomarker (cTnI for MI patients) and sensitive detection of biomarker (LOD of 0.01 ng mL^−1^). For selection of biomarker, the cTnI was a better biomarker for MI patients due to the characteristic release of cTnI from myocardial cell to blood, compared with other biomarkers (e.g., CK-MB), according to literature reports^47,48^. For detection of biomarker, sensitive detection of cTnI with LOD of 0.01 ng mL^−1^ may lead to the clear differentiation between early MI patients with higher cTnI concentrations and healthy controls with lower cTnI concentrations^49,50^, superior to other platforms (e.g., glass chips and sGold chips) with lower detection sensitivity. Therefore, we identified healthy controls with lower cTnI concentrations at high confidence (specificity of >95%) by the rational selection and sensitive detection of the biomarker.

### Speed and multiplexity of pGold chip assay

**Fig. 4:**
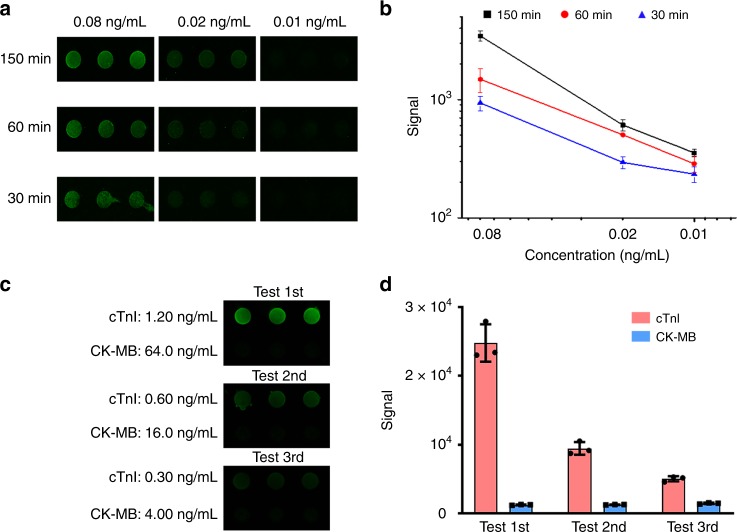
Control of reaction time and cross reactivity tests. **a** Fluorescence mapping results and **b** corresponding calibration curves with the reaction time of 150, 60, and 30 min for cTnI (0.01–0.08 ng mL^−1^). All experiments were conducted with *n* = 3; mean ± s.d. **c** Fluorescence images showing only one row of spots (cTnI) emitting bright fluorescence signals when a multiplexed antibody chip (two rows, two different antibodies against biomarkers labeled at the left of the image with different concentrations) were incubated in a solution containing a mixture of the two antigens, followed by incubation in a solution containing only one of the corresponding detection antibodies without the other one. **d** Averaged fluorescence intensity of each row in (**c**). All experiments were conducted with *n* = 3; mean ± s.d. Source data are provided as a Source Data file.

Considering emergencies in real case calling for fast diagnosis of MI, the speed of the pGold chip assay was investigated. NIR-FE detection of cTnI was performed at low concentrations of 0.01–0.08 ng mL^−1^ (Fig. 4a), with controlled overall immuno-reaction time of 150/60/30 min on-chip. The corresponding calibration curves displayed reduced NIR fluorescence intensities by 15–50% (Fig. 4b). Notably, the LOD and LOQ reached 0.0152 and 0.0331 ng mL^−1^, based on 30 min immuno-reaction on chip (Supplementary Table 7). It has been anticipated that with further instrumentation and automation, our pGold chip assay can engage the requirement for the diagnosis of MI in a much shorter time for emergencies.

Also, multiplex detection of biomarkers was conducted on the pGold chips. Capture antibodies for cTnI and CK-MB were applied in a 3 × 2 spot matrix format with each row containing one type of capture antibodies. For specificity tests, we implemented one detection antibody for biomarkers with different concentrations (0.3–1.2 ng mL^−1^ for cTnI, 4.0–64.0 ng mL^−1^ for CK-MB) and observed bright fluorescent spots in the target row only (Fig. 4c, Supplementary Fig. 5a). Averaged fluorescence intensities of spots in each row were summarized in Fig. 4d, Supplementary Fig. 5b. These data demonstrated the capability of pGold chip assay to carry out multiplex biomarker analysis.

### Post-operative monitoring of MI patients after PCI

**Fig. 5:**
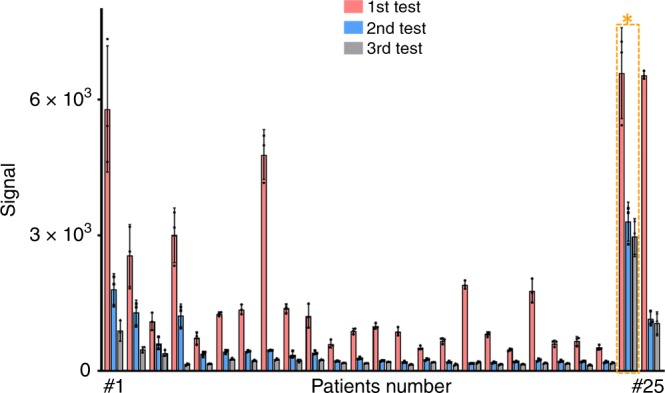
Serum tests for monitoring after PCI using cTnI. Tri-tests of cTnI for all 25 patients in a period of 10 days, concluding 1st test results before PCI in red, 2nd test results on day 5 after PCI in black and 3rd test results on day 10 after PCI in blue. All experiments were conducted with *n* = 3; mean ± s.d. * referred to the MI patient (#24, in the yellow box) that needed observation for a longer time after PCI. Source data are provided as a Source Data file.

Further, the serum concentrations of cTnI were monitored to check the health conditions of MI patients after PCI (Fig. 5). A cohort of 25 MI patients (19 males and 6 females) were analyzed with a median age of 68 (41–92). For past medical history, 5 patients had high blood pressure, 14 patients had no related diseases, and 6 patients had MI within recent 3 years (Supplementary Table 8). Three blood samples per patient were collected and monitored including one blood sample before PCI and two blood samples within 10 days (one on day 5 and the other on day 10) after PCI. Specifically, it was found that serum concentrations of cTnI gradually decreased for the subsequent two tests after PCI for MI patients (*p* < 0.05), compared with levels of cTnI before PCI (*p* < 0.0001). The gradual decrease of cTnI concentrations can be obtained in serum for all the 25 patients in three consecutive tests for short-term (10 days) outcome assessment that would be relevant to long-term (from 3 months to 8 years) morbidity and mortality for potential prognostic use^51–54^.

PCI with around millions of cases per year (known as a preferred method of myocardial revascularization), is a commonly used technique for treating MI^55^. Notably, monitoring of PCI is crucial and decides the life quality for MI patients, while the cTnI level in serum serves as the main indicator in clinics. Considering the key role of cTnI and application benefit of our assay, the pGold chip assay may help to address the technical challenges and application needs dealing with MI from a clinical perspective, toward efficient detection and monitoring of MI in daily life.

To better put our findings into perspective, we included a small cohort of 10 patients (Supplementary Fig. 6 and Supplementary Table 9) presenting with acute chest pain to the emergency department of Shanghai Chest Hospital (intended use Population) and adjudicated the final diagnosis according to the universal definition using serial measurements of cTnI by pGold. In monitoring of cTnI, five patients (No. 1–5) with acute chest pain showed a significant elevation (≥20%, according to the literature)^56,57^ between two consecutive time points (0 h/1 h and 0 h/3 h), who were diagnosed with MI (in conjunction with clinical history, physical examination, and ECG) and required intervention treatment. For comparison, another five patients (No. 6–10) with acute chest pain showed no significant elevation between two consecutive time points (0 h/1 h and 0 h/3 h), who were diagnosed without MI (in conjunction with clinical history, physical examination, and ECG) and required no intervention treatment.

Our approach is different from previous reports owing to two major aspects, including disease diagnostics and application design. For disease diagnosis, we reported the assay based on plasmonic nanochips for diagnosis of myocardial infarction, distinct from previous reports using the similar plasmonic platform, such as diagnosis of type I child diabetes^23^ and Zika virus infection^26^. For application design, this work contained the analytical features (compared with previous reports)^58,59^ as tailored, including multi-functions (monitoring for prognostic use) in clinics, selection of specific biomarkers (cTnI and CK-MB), and low sample consumption in high speed (10 μL of serum for 30 min reaction). In addition, our plasmonic gold nano-island platform afforded enhancement factors of ~130-fold, compared with the typical literatures^58,59^ with optimized enhancement factors of 50-fold and 99-fold using plasmonic gold nanoparticles. Therefore, our approach addressed the application needs and technical challenges from a clinical perspective, different from typical literatures^58,59^ on sensing mechanism and device structure using plasmonic nanostructures to enhance the fluorescence of dyes from pure chemical/physical perspective.

In summary, we introduced pGold chips as a platform in the management of MI. Our approach featured for (1) quantitation of selected biomarkers (e.g., cTnI) at low concentrations down to 0.01 ng mL^−1^; (2) capability for detection of trace serum samples (10 μL) toward POCT; (3) diagnosis of MI with the optimized sensitivity of 100% and specificity of 95.54%; and (4) desirable speed and multiplexity for diagnostics and monitoring for prognostic use. This work contributed to the design of materials/device based POCT and the development of precision medicine for diverse diseases with tailored characteristics in near future.

## Methods

### Preparation of chips

Three types of chips were used, including glass chips, sputtered gold (sGold) chips, and plasmonic gold nano-island (pGold) chips. Glass chips with epoxy group were obtained from CapitalBio Company, China. pGold chips with gold nano-islands were obtained from Nirmidas Biotech Inc. and sGold chips were prepared by the magnetic sputtering method^28^. Glass slides were sputtered with gold using a magnetic control sputtering coater (GSL-1100X-SPC-16M) from Shenyang Kejing Auto-instrument Co., Ltd, China. Sputtering process was carried out at 40 mA current for 4 min under vacuum. Gold plate (99.99%) for sputtering was ordered from Shanghai Daheng Optical Precision Machinery CO., LTD. Glass slides were immersed in chloroauric acid solution at the concentration of 3 × 10^−3^ M. NH_4_OH was added by the volume ratio of 1:50 with rapid shaking for 1 min and immerged into deionized water for washing. Then slides were put into sodium borohydride solution at a concentration of 1 × 10^−3^ M for 1 min and washed with deionized water again. Finally, the slides were shifted to chloroauric acid solution and hydroxylamine solution at the mole ratio of 1:1 for 5 min of shaking and 10 min of incubation. Slides were dried after washing and stored in a sealable container.

### Construction of device

Device was built in a manner for easy assembling. FAST frame slide holder and incubation chamber were ordered from Grace Bio-Labs, America. First, the chip was embedded into the incubation chamber and divided into 16 independent reaction wells. Then installed chip was loaded into FAST frame slide holder along two side grooves. The assembled device can be used to conduct immunoassays.

### Microarray printing

Nano-Plotter TM 2.1 (GeSim Co., Germany) was applied to immobilize the capture antibodies on the chips (including glass, sGold, and pGold chips). Chips with immobilized antibodies were prepared by the non-contact micro-dispensing method. Capture antibodies were diluted with 1 × phosphate buffer saline (PBS, pH = 7.4) to the concentration of 3 μM. Then capture antibodies were printed in triplicate with each spot volume of 3 nL by running the designed printing program. The diameter of each spot was about 400 μm. The chip was placed at 4 °C before use.

### Characterization

For characterizations of chips, the extinction spectra of various chips were recorded with a UV1900 UV-Vis spectrometer (Aucybest, China). Scanning electron microscopy (SEM) images were acquired on Hitachi S-4800 with field emission gun (FEG) source at acceleration voltage of 10.0 kV. Digital images were recorded by iPhone 7.

### Antibody labeling by IRDye800

Detection antibodies were labeled with IRDye800 though 1-ethyl-3-(3-dimethyl aminopropyl) carbodiimide/N-hydroxysuccinimide (EDC-NHS) conjugation. Illustra NAP-5 columns were ordered from GE Healthcare and IRDye 800CW N-hydroxysuccinimide (NHS) ester was purchased from LI-COR Biosciences. IRDye 800CW NHS ester was diluted with dimethyl sulphoxide (DMSO) and detection antibody was mixed at the mole ratio of 1:4 and shaking for 1.5 h in the dark. First, 10 mL of 1 × PBS buffer (pH = 7.4) was added into the column. Then the mixture was added with 1 × PBS buffer (pH = 7.4) to make the total volume of 500 μL after dripping. Finally, IRDye800 labeled detection antibody solution was collected with 500 μL of 1 × PBS buffer (pH = 7.4) and stored at −20 °C in dark before use.

### Harvest of serum samples

All of the investigation protocols in this study were approved by the institutional ethics committees of the Shanghai Chest Hospital, Shanghai Jiao Tong University (Reference No. KS(P)1703). All serum samples detected in the experiment were collected in Shanghai Chest Hospital according to the established protocol^60^. Three milliliters of blood was drawn to a serum tube and centrifuged at 3000 rpm for 15 min. Aliquots of supernatant were collected and stored at −20 °C before use.

### Immunoassay on chip

A sandwich structure was applied to detect serum biomarkers. Capture antibodies for cTnI (cat. # 14T21, mAb: M18)/CK-MB (mAb: 1C11), Detection antibodies for cTnI (cat. # 14T21, mAb: 19C7)/CK-MB (mAb: 1D10), and calibration materials for cTnI/CK-MB were obtained from Shanghai Tellgen Life Science Company. Printed chips (including glass, sGold, and pGold chips) were blocked with 5% bovine serum albumin (BSA) solution (diluted with 1 × PBS buffer containing 0.05% tween-20) for 1 h shaking to reduce non-specific binding. Then, 150 μL of 1 × PBS buffer containing 0.05% tween-20 (PBST) was used to wash each reaction well. For calibration curves detection, calibration samples containing known concentrations of cTnI and CK-MB were diluted in pure water as the kit (Tellgen) suggested to a certain concentration. For serum samples detection, 10  μL of human serum was diluted into 90 μL of 10% fetal bovine serum (FBS) solution. Hundred microliters of solution from calibration sample and serum sample were added in each reaction well for 120 min of shaking at room temperature. After washing with PBST for five times, the chip was then stained IRDye800 labeled antibodies for anti-cTnI and anti-CK-MB at a concentration of 10 × 10^−9^ M for 30 min shaking in the dark. Obtained chips were washed three times by PBST. Hundred microliters of PBST and PBS were added into each reaction well for 15 min and 5 min shaking in the dark, subsequently. The chips were finally washed with deionized water and dried with compressed air before scanning.

### NIR fluorescence measurement

The chips were scanned by InnoScan 710-IR microarray scanner using the 785 nm channel with a resolution of 10 μm per pixel. A 16-bit grayscale image can be exported after scanning. Then images were analyzed by Image Studio Version 3.1.4. Spot features were automatically identified by the program and then manually defined^22^. The intensity of each feature was calculated as the total signal divided by the area. The mean intensity of the three spots was used for quantitation.

### Chemiluminescence immunoassay

Chemiluminescence immunoassay (CIA) was conducted on the UniCel DxI 800 (Beckman Coulter Inc.) using both 200 μL (for the standard of care) and 10 μL of serum. The CIA on the UniCel DxI 800 platform (Beckman Coulter) is a reported high-sensitivity cTnI assay in clinic use^61–63^ and requires 200 μL of serum for the optimal standard of care use to make the clinical diagnosis. Also, CIA based on one high-sensitivity cTnI assay (as recommended in the current guidelines^37,40,46^) was conducted using Abbott Architect kit (Abbott Architect STAT high-sensitivity cTnI reagent kit) and i2000SR platform (consuming 200 μL of serum). For calibration curves, standard samples (containing known concentrations of cTnI/CK-MB) were re-dissolved by pure water. In serum analysis, 10 μL of human serum was diluted into 90 μL of 10% FBS solution (same as the gGold chip assay). Standard or serum samples were mixed with detection antibody for cTnI/CK-MB (conjugated with alkaline phosphatase) in a surfactant-containing buffer. Then magnetic particles with capture antibodies were added. After incubation, magnetic particles were collected to measure the fluorescence based on the chemiluminescent substrate Lumi-Phos* 530 for quantitation.

### LODs, LOQs, and cutoffs

LODs and LOQs were calculated for cTnI and CK-MB, according to the calibration curves by the pGold chips and CIA. The linear fitting equation of each calibration curve of two platforms was computed by OriginPro 8. LOD was measured as the mean blank value plus 3 times of s.d. and LOQ was measured as the mean blank value plus 10 times of s.d. The intensity cutoffs were corresponding to serum concentrations of 0.04 ng mL^−1^ (recommended cutoff by CIA) and 0.03 ng mL^−1^ (lowered cutoff by pGold) for cTnI, 4 ng mL^−1^ (recommended cutoff by CIA) and 3.13 ng mL^−1^ (lowered cutoff by pGold) for CK-MB.

### Diagnostic and monitoring experiments

Myocardial infarction (MI) was defined as pathologically as the death of myocardial cell due to the prolonged ischaemia^57^, which can be diagnosed on the basis of the 99th percentile of cTnI, clinical manifestation, ECG, and angiography according to the literature reports^40,57^. A case-control design was used and the 99th percentile of cTnI can be obtained using controls. The blood samples were obtained during an early screening of initial MI patients with chest pain and without treatment, who were later diagnosed with MI on the basis of the 99th percentile of cTnI, clinical manifestation, ECG, and angiography^40,57^. The cTnI concentration representing the 99th percentile of the overall study population was 0.036 ng mL^−1^ (36 ng L^−1^), consistent with the cutoffs of the current high-sensitivity cTnI assays (14–52 ng L^−1^). An elevation above the 99th percentile of cTnI is a mandatory criteria for the diagnosis of MI in vivo.

Typically, MI patients in China would first take blood tests for early screening purpose, then have coronary angiography for validation and potential percutaneous coronary intervention (PCI), according to the appropriate use criteria for coronary revascularization as reported^64,65^. For diagnostic application, MI serum samples were from 112 MI patients (75 males and 37 females) with a median age of 64.39, who were diagnosed without malignant tumor, autoimmune disorders, severe infectious diseases, trauma, heart diseases, and other major diseases. Control serum samples were collected from 112 healthy controls (81 males and 31 females) with a median age of 61.66, who were diagnosed without MI and other major diseases. Patients/controls known to have other medical conditions (such as active bleeding) were excluded. Both age and gender were matched with *p* > 0.05 (according to Student’s *t*-test and Fisher’s exact test), between the MI patients and healthy controls.

For monitoring application, a cohort of 25 MI patients (19 males and 6 females) were analyzed with a median age of 68 (41–92). For past medical history, 5 patients had high blood pressure, 14 patients had no related diseases, and 6 patients had MI within recent 3 years. Three blood samples per patient were collected and monitored including one blood sample before PCI and two blood samples within 10 days (one on day 5 and the other on day 10) after PCI. Monitoring concentrations of cTnI after PCI can be critical in short-term (10 days in this work) outcome assessment that would be relevant to long-term (from 3 months to 8 years) morbidity and mortality according to literature reports^51–54^. Also, a small cohort of 10 patients presenting with acute chest pain to the emergency department of Shanghai Chest Hospital (intended use Population) was added and the final diagnosis was adjudicated according to the universal definition using serial measurements.

### Control of reaction time

Reaction time of immunoassay was controlled to be 150, 60, and 30 min on pGold chips, including the capture and detection steps. Specifically, in the capture step, calibration samples containing known concentrations of cTnI (0.08, 0.02, and 0.01 ng mL^−1^) were incubated for 120, 40, and 20 min, individually. In the detection step, antibody staining (by 2 nM IRDye800 labeled detection antibodies) time was set to be 30, 20, and 10 min, individually.

### Cross reactivity tests

The procedure was the same with standards or serum detection on pGold chips except for the detection step. Calibration samples containing a mixture of cTnI (0.3–1.2 ng mL^−1^) and CK-MB (4.0–64.0 ng mL^−1^) were applied to the pGold chips. In the detection step, one group of wells was added with only one IRDye800 labeled antibody, another group of wells added with the other antibody instead.

### Statistical analysis

Coefficient variation was calculated as standard deviation divided by mean intensity. Positive cases among patient samples and negative cases among control samples were used to calculate the sensitivity (as true positive/(true positive + false negative)) and specificity (as true negative/(true negative + false positive)). Statistical Product and Service Solutions 19 (SPSS 19.0, SPSS Inc., Chicago) was used to plot and analyze the ROC curve. The CI and *p*-value were calculated using the SPSS software based on the Clopper–Pearson method, Fisher’s exact test, unpaired Student’s *t*-test, and Wilcoxon signed-rank test^66–69^.

### Reporting summary

Further information on research design is available in the Nature Research Reporting Summary linked to this article.

## Supplementary information

Supplementary Information

Reporting Summary

## Data Availability

The datasets obtained and analyzed during the current study are available from the corresponding authors upon reasonable request. The source data for Figs. 1b, 2b, c, e, f, 3a, c, 4b, d, and 5, and Supplementary Figs. 2a, b, 3, 4, 5b, and 6 were provided as a Source Data file.

## Source Data

Source Data
