# Supplementary material for: Diagnosis and prognosis of myocardial infarction on a plasmonic chip
Source: Nat Commun. 2020 Apr 3;11:1654. doi: 10.1038/s41467-020-15487-3 (PMC7125217; doi:10.1038/s41467-020-15487-3)
Supplement: Supplementary file 1 — Supplementary Information [file 41467_2020_15487_MOESM1_ESM.pdf]

Supplementary Information

Diagnosis and prognosis of myocardial infarction on a plasmonic chip

Xu et al.

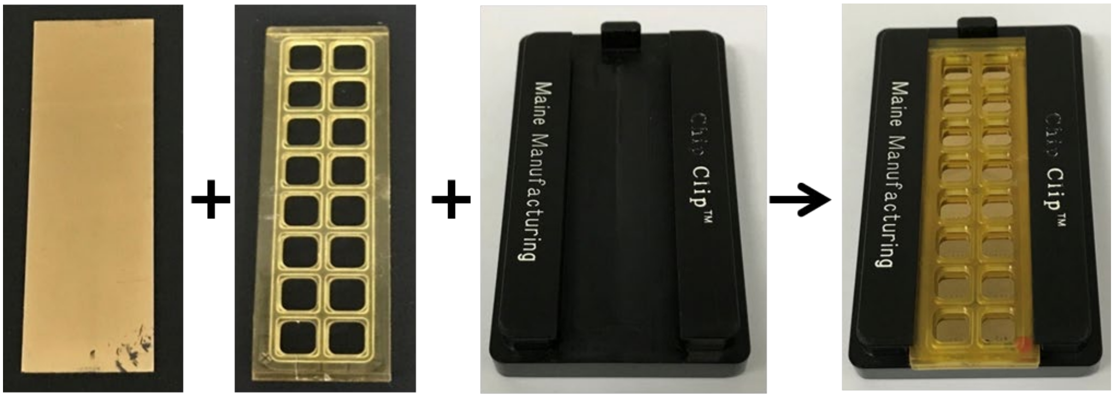

a b c d

Supplementary Fig. 1. **Digital photographs.** a) pGold chip, b) incubation chamber, and c) FAST frame slide holder was assembled to d) device for immunoassay.

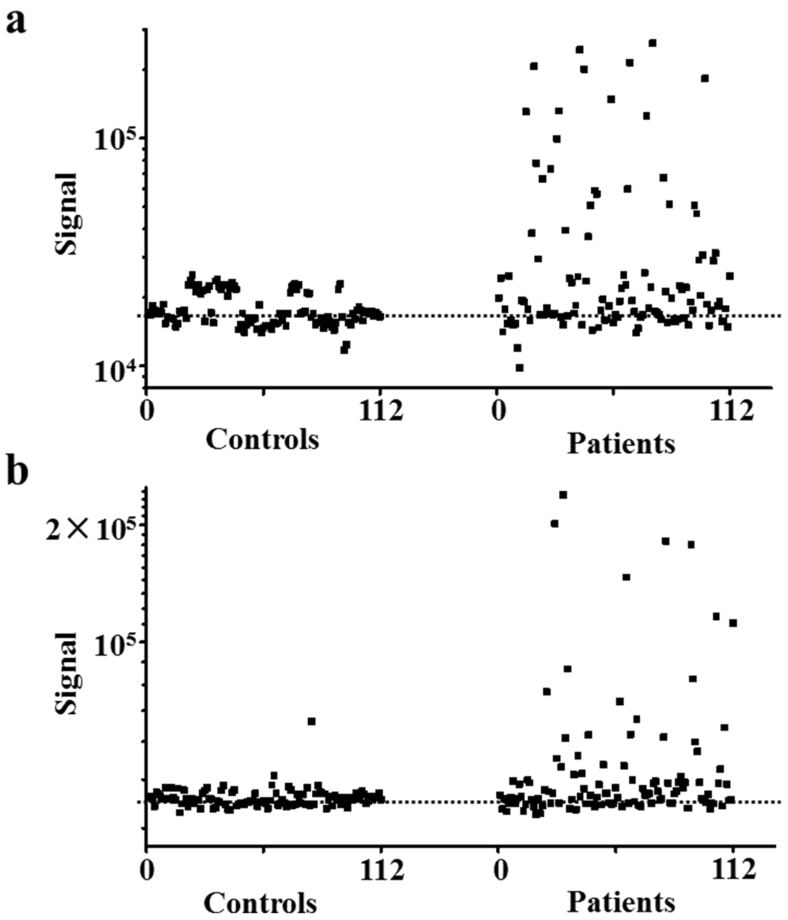

Supplementary Fig. 2. **Serum tests for diagnosis of MI by CIA.** a) Signal quantification for the detection of MI patients and controls using cTnI. b) Signal quantification for the detection of MI patients and controls using CK-MB. The dashed lines represented the intensity cutoffs corresponding to serum concentrations of 0.03 ng mL^{-1} for cTnI and 3.13 ng mL^{-1} for CK-MB. n = 224 independent serum samples examined over 1 experiment. Source data are provided as a Source Data file.

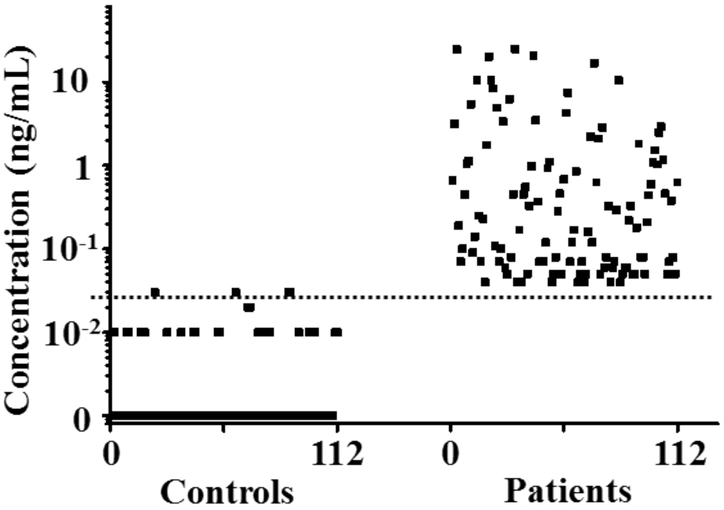

Supplementary Fig. 3. Serum tests for diagnosis of MI using the high-sensitivity cTnI assay (consuming 200 μL of serum). The dashed line represented the cutoff concentration of cTnI corresponding to 0.03 ng mL^{-1} in serum. n = 224 independent serum samples examined over 1 experiment. Source data are provided as a Source Data file.

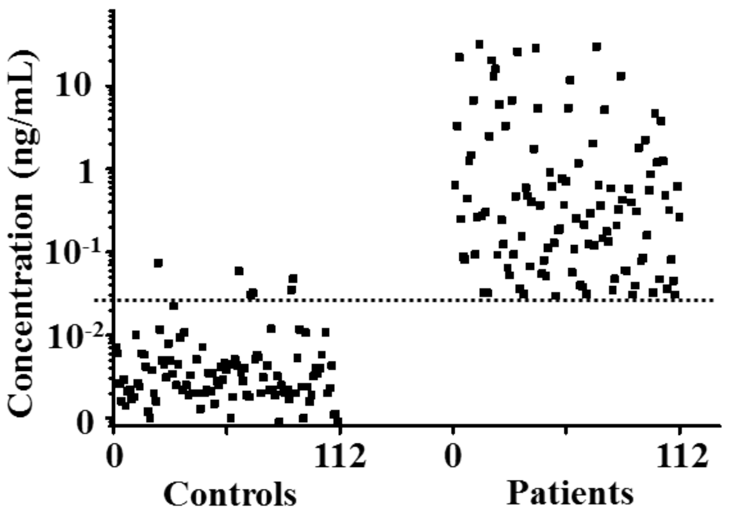

Supplementary Fig. 4. Serum tests for diagnosis of MI using the Abbott Architect kit on i2000SR platform. The cTnI concentrations in serum samples were measured using the Abbott Architect kit on i2000SR platform. The dashed line represented the cutoff concentration of cTnI corresponding to 0.03 ng mL^{-1} in serum. n = 224 independent serum samples examined over 1 experiment. Source data are provided as a Source Data file.

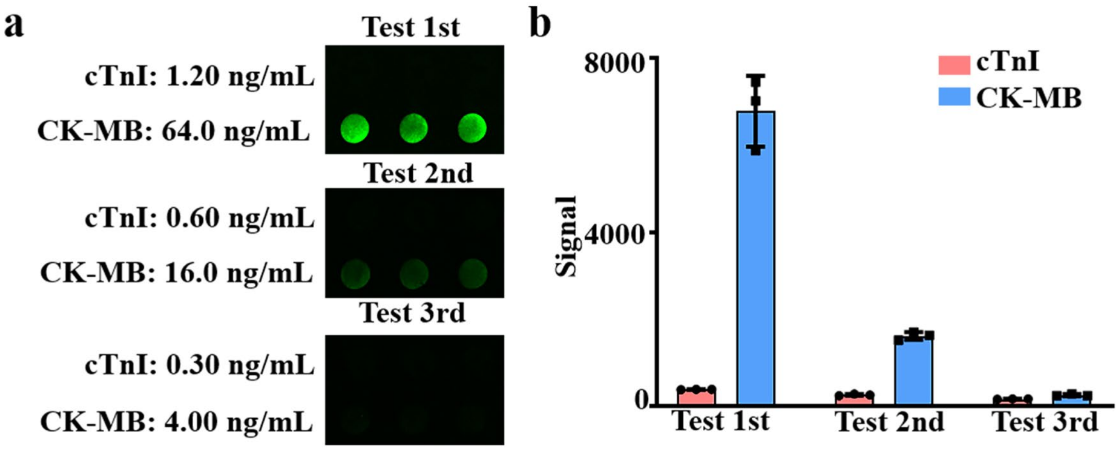

Supplementary Fig. 5. **Cross reactivity tests.** a) Fluorescence images showing only one row of spots (CK-MB) emitting bright fluorescence signals when a multiplexed antibody chip (two rows, two different antibodies against biomarkers labeled at the left of the image with different concentrations) were incubated in a solution containing a mixture of the two antigens, followed by incubation in a solution containing only one of the corresponding detection antibodies without the other one. b) Averaged fluorescence intensity of each row in a). All experiments were conducted with n = 3; mean ± s.d.. Source data are provided as a Source Data file.

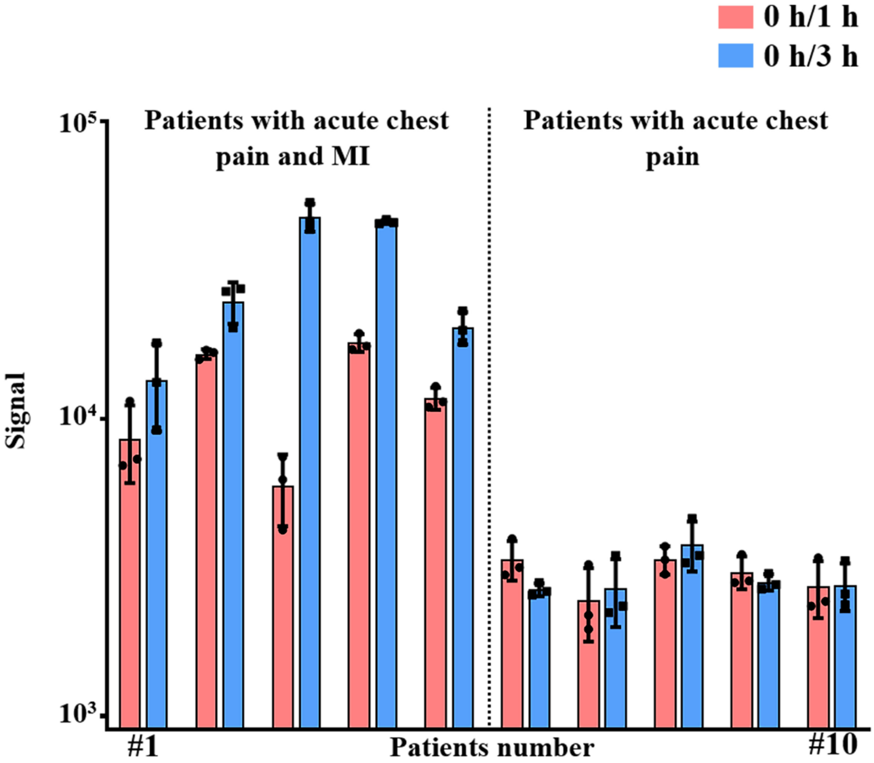

Supplementary Fig. 6. Serum tests for monitoring using cTnI in a group of 10 patients with acute chest pain. 5 patients (No. 1-5) showed a significant elevation (≥ 20%) between two consecutive time points (0 h/1 h and 0 h/3 h), who were diagnosed with MI (in conjunction with clinical history, physical examination, and ECG) and required intervention treatment. Another 5 patients (No. 6-10) showed no significant elevation between two consecutive time points (0 h/1 h and 0 h/3 h), who were diagnosed without MI (in conjunction with clinical history, physical examination, and ECG) and required no intervention treatment. Error bars represented the standard deviation of the triplicate spots for each test. All experiments were conducted with n = 3; mean ± s.d.. Source data are provided as a Source Data file.

Supplementary Table 1. The average fluorescence intensity of different concentrations of cTnI by pGold chips, glass chips, sGold chips, and CIA.

Concentration [ng mL^{-1}]	pGold	Glass	sGold	CIA
1.2	17478.84	510.32	74.91	537510
0.3	5915.44	119.48	74.21	117315
0.08	3564.45	84.09	73.60	37508
0.04	1263.08	76.38	73.65	17545
0.02	591.09	75.86	74.50	14924
0.01	380.76	77.48	75.70	12632
blank	188.22	75.64	74.44	11822

Supplementary Table 2. Summary of average fluorescence intensity enhancement fold analysis of cTnI and CK-MB.

	Background signal[a]	Fluorescence Intensity[b]	Direct enhancement (fold)[c]	Subtracted enhancement (fold)[d]
cTnI (0.3 ng mL^{-1}) on glass chip	75.64	119.48	/	/
CK-MB (64 ng mL^{-1}) on glass chip	76.67	176.18	/	/
cTnI (0.3 ng mL^{-1}) on pGold chip	188.22	5915.44	49.5	130.7
CK-MB (64 ng mL^{-1}) on pGold chip	120.55	6529.01	37.1	64.4

a) Average background signal intensity; b) Average fluorescence intensity; c) Direct enhancement folds of fluorescence signals on the pGold chips against glass chips; d) Subtracted enhancement folds of fluorescence signals on the pGold chips against glass chips.

Supplementary Table 3. The average fluorescence intensity of different concentrations of CK-MB by pGold chips, glass chips, sGold chips, and CIA.

Concentration [ng mL^{-1}]	pGold	Glass	sGold	CIA
64	6529.01	176.18	81.86	508712
16	1984.73	115.96	74.10	119042
4.0	412.79	79.02	75.88	44418
1.0	182.82	76.89	74.77	32575
0.25	135.36	77.70	74.77	23085
blank	120.55	76.67	73.08	20498

Supplementary Table 4. Coefficient variations for different concentrations of cTnI by pGold chips and CIA.

Concentration [ng mL^{-1}]	pGold[a] (%)	CIA[a] (%)
1.2	5.52	3.53
0.3	14.53	8.53
0.08	4.20	5.79
0.04	11.87	4.68
0.02	12.05	3.84
0.01	2.91	3.46
blank	13.01	2.41

a) Coefficient variation was calculated as the standard deviation of the three independent tests divided by mean intensity.

Supplementary Table 5. Coefficient variations for different concentrations of CK-MB by pGold chips and CIA.

Concentration [ng mL^{-1}]	pGold[a] (%)	CIA[a] (%)
64	6.48	0.14
16	10.62	3.60
4.0	9.00	1.72
1.0	14.17	10.14
0.25	8.43	5.43
blank	10.31	8.06

a) Coefficient variation was calculated as the standard deviation of the three independent tests divided by mean intensity.

Supplementary Table 6. Clinical subjects for diagnosis (112 patients and 112 controls).

Subjects	Number	Gender		p value[a]	Age (median/range)	p value[b]
		Female	Male			
Controls	112	31	81	0.468	61.66 (39-84)	0.297
Patients	112	37	75		64.39 (40-87)	

a) p value was calculated based on Fisher's exact test. b) p value was calculated based on the two-sided Student's t-test.

Supplementary Table 7. LODs and LOQs for different reaction time by pGold chip.

	pGold - LOD[a]	pGold - LOQ[b]
cTnI (150 min)	0.0082	0.0136
cTnI (60 min)	0.0093	0.0185
cTnI (30 min)	0.0152	0.0331

a) LODs were calculated by applying the mean blank value plus 3 times of s.d. to the fitting line of calibration curves; b) LOQs were calculated by applying the mean blank value plus 10 times of s.d. to the fitting line of calibration curves.

Supplementary Table 8. Clinical subjects for monitoring (25 patients).

Patient number	Sex	Gender	Medical history	PCI
1	Male	82	MI	Yes
2	Male	68	MI	Yes
3	Female	65	NA	Yes
4	Male	70	NA	Yes
5	Female	68	HBP	Yes
6	Male	77	NA	Yes
7	Female	67	NA	Yes
8	Male	84	HBP	Yes
9	Male	70	NA	Yes
10	Male	58	MI	Yes
11	Male	58	NA	Yes
12	Male	63	HBP	Yes
13	Male	52	NA	Yes
14	Male	41	MI	Yes
15	Female	59	NA	Yes
16	Female	44	NA	Yes
17	Male	36	NA	Yes
18	Female	92	MI	Yes
19	Male	71	HBP	Yes
20	Male	67	NA	Yes
21	Male	67	NA	Yes
22	Male	69	HBP	Yes
23	Male	79	MI	Yes
24	Male	76	NA	Yes
25	Male	78	MI	Yes

MI: Myocardial infarction; NA: No other related diseases; HBP: High blood pressure; PCI: percutaneous coronary intervention.

Supplementary Table 9. Clinical subjects with acute chest pain.

Patient number	Sex	Gender	Diseases
1	Male	62	MI
2	Female	55	MI
3	Male	82	MI
4	Male	79	MI
5	Male	67	MI
6	Female	71	CAD
7	Male	51	CAD
8	Male	72	CAD
9	Male	78	CAD
10	Male	78	CAD

MI: Myocardial infarction; CAD: Chronic coronary artery disease.
