# Supplementary material for: Diagnosis and prognosis of myocardial infarction on a plasmonic chip
Source: Nat Commun. 2020 Apr 3;11:1654. doi: 10.1038/s41467-020-15487-3 (PMC7125217; doi:10.1038/s41467-020-15487-3)
Supplement: Supplementary file 2 — Reporting Summary [file 41467_2020_15487_MOESM2_ESM.pdf]

nature research

Corresponding author(s): Kun Qian
Last updated by author(s): Mar 2, 2020

Reporting Summary

Nature Research wishes to improve the reproducibility of the work that we publish. This form provides structure for consistency and transparency in reporting. For further information on Nature Research policies, see Authors & Referees and the Editorial Policy Checklist.

Statistics

For all statistical analyses, confirm that the following items are present in the figure legend, table legend, main text, or Methods section.

n/a	Confirmed	
☐	☒	The exact sample size (*n*) for each experimental group/condition, given as a discrete number and unit of measurement
☐	☒	A statement on whether measurements were taken from distinct samples or whether the same sample was measured repeatedly
☐	☒	The statistical test(s) used AND whether they are one- or two-sided<br>*Only common tests should be described solely by name; describe more complex techniques in the Methods section.*
☐	☒	A description of all covariates tested
☐	☒	A description of any assumptions or corrections, such as tests of normality and adjustment for multiple comparisons
☐	☒	A full description of the statistical parameters including central tendency (e.g. means) or other basic estimates (e.g. regression coefficient) AND variation (e.g. standard deviation) or associated estimates of uncertainty (e.g. confidence intervals)
☐	☒	For null hypothesis testing, the test statistic (e.g. *F*, *t*, *r*) with confidence intervals, effect sizes, degrees of freedom and *P* value noted<br>*Give P values as exact values whenever suitable.*
☒	☐	For Bayesian analysis, information on the choice of priors and Markov chain Monte Carlo settings
☒	☐	For hierarchical and complex designs, identification of the appropriate level for tests and full reporting of outcomes
☐	☒	Estimates of effect sizes (e.g. Cohen's *d*, Pearson's *r*), indicating how they were calculated

Our web collection on statistics for biologists contains articles on many of the points above.

Software and code

Policy information about availability of computer code

Data collection	Image Studio Version 3.1.4
Data analysis	SPSS software (version 19.0)<br>Origin (version 8.0)

For manuscripts utilizing custom algorithms or software that are central to the research but not yet described in published literature, software must be made available to editors/reviewers. We strongly encourage code deposition in a community repository (e.g. GitHub). See the Nature Research guidelines for submitting code & software for further information.

Data

Policy information about availability of data

All manuscripts must include a data availability statement. This statement should provide the following information, where applicable:

- Accession codes, unique identifiers, or web links for publicly available datasets
- A list of figures that have associated raw data
- A description of any restrictions on data availability

The datasets obtained and analyzed during the current study are available from the corresponding authors upon reasonable request. The source data for Figs. 1b, 2b-c, e-f, 3a&c, 4b&d, and 5, and Supplementary Figs. 2a-b, 3, 4, 5b, and 6 were provided as a Source Data file.

Field-specific reporting

Please select the one below that is the best fit for your research. If you are not sure, read the appropriate sections before making your selection.

☒ Life sciences ☐ Behavioural & social sciences ☐ Ecological, evolutionary & environmental sciences

For a reference copy of the document with all sections, see nature.com/documents/nr-reporting-summary-flat.pdf

Life sciences study design

All studies must disclose on these points even when the disclosure is negative.

Sample size	We estimated the sufficient sample size with one-sample sensitivity and specificity analysis (sensitivity=0.9, specificity=0.9).
Data exclusions	No data were excluded from our analyses.
Replication	We performed three independent experiments and data are shown as the mean ± s.d. (n=3)
Randomization	The participants were allocated randomly.
Blinding	The pathologists were blind to participants' clinical information and any other information about the acquisition results from plasmonic gold nano-island (pGold) chip detection.

Reporting for specific materials, systems and methods

We require information from authors about some types of materials, experimental systems and methods used in many studies. Here, indicate whether each material, system or method listed is relevant to your study. If you are not sure if a list item applies to your research, read the appropriate section before selecting a response.

Materials & experimental systems

n/a	Involved in the study	
☐	☒	Antibodies
☒	☐	Eukaryotic cell lines
☒	☐	Palaeontology
☒	☐	Animals and other organisms
☐	☒	Human research participants
☒	☐	Clinical data

Methods

n/a	Involved in the study	
☒	☐	ChIP-seq
☒	☐	Flow cytometry
☒	☐	MRI-based neuroimaging

Antibodies

Antibodies used	Capture antibodies for cTnI(cat. # 14T21, mAb: M18, mouse, 3 μM), for CK-MB(mAb: 1C11, mouse, 3 μM),<br>Detection antibodies for cTnI(cat. # 14T21, mAb: 19C7, mouse, 10 × 10-9 M), for CK-MB(mAb: 1D10, mouse, 10 × 10-9 M)
Validation	Detection and capture antibodies for cTnI/CK-MB were obtained from Shanghai Tellgen Life Science Company.<br>Detection and capture antibodies for cTnI were also quality control tested and analyzed by HyTest (Shanghai) Ltd. (https://www.hytest.fi/home)<br>Detection and capture antibodies for CK-MB were also quality control tested and analyzed by Fapon Biotech Inc. (http://en.diagnostics.fapon.com/)

Human research participants

Policy information about studies involving human research participants

Population characteristics	For diagnostic application, MI serum samples were from 112 MI patients (75 males and 37 females) with a median age of 64.39, who were diagnosed without malignant tumor, autoimmune disorders, severe infectious diseases, trauma, heart diseases, and other major diseases. Control serum samples were collected from 112 healthy controls (81 males and 31 females) with a median age of 61.66, who were diagnosed without MI and other major diseases. No significant age difference was observed among all groups (p=0.297 by Student's t-test). Gender was also matched for controls and patients (p=0.468 by Fisher's exact test). Patients/controls known to have other medical conditions (such as active bleeding) were excluded.
Recruitment	Subjects were consecutively recruited in Shanghai Chest Hospital. All patients were diagnosed by the 99th percentile of cTnI, clinical manifestation, ECG, and angiography according to the current NSTE-ACS ESC guidelines. The pathologists were blind to any information about the acquisition results from plasmonic gold nano-island (pGold) chip detection. Patients were excluded

from the study if they were diagnosed with malignant tumor, autoimmune disorders, severe infectious diseases, trauma, heart diseases, and other major diseases.

Ethics oversight	All of the investigation protocols in this study were approved by the institutional ethics committees of the Shanghai Chest Hospital, Shanghai Jiao Tong University (reference No. KS(P)1703).

Note that full information on the approval of the study protocol must also be provided in the manuscript.
